# Where are the gaps in improving maternal and child health in Mauritania? the case for contextualised interventions: a cross sectional study

**DOI:** 10.11604/pamj.2013.14.97.2292

**Published:** 2013-03-11

**Authors:** Frédérique Vallières, Emma Louise Cassidy, Eilish McAuliffe, Sidina Ould Isselmou, Mohamed Saleh Hamahoullah, Juliet Lang

**Affiliations:** 1 Centre for Global Health, Trinity College Dublin, 7-9 Leinster Street South, Dublin 2, Ireland; 2 World Vision Mauritania, ZRB 510 Tevragh Zeina M/S, Nouakchott, BP 335 Mauritanie; 3 World Vision Ireland, The Mews, Rathmines Park, Dublin 6, Ireland

**Keywords:** maternal and child health, West Africa, MDGs, Mauritania, rural

## Abstract

**Introduction:**

It is estimated that any progress made towards improving maternal and child health in Mauritania has likely stalled. A lack of reliable and up-to-date data regarding maternal and child health indicators makes it difficult to identify current gaps and adapt international programmes to meet local needs.

**Methods:**

Using secondary data collected as part of a baseline assessment for a maternal and child health programme being implemented in two health departments, we compared maternal and child health indicators across two different samples of pregnant women and children under-five in M'bagne and Guérou. Descriptive analyses were conducted using a Pearson's Chi-Squared test, assuming a binomial distribution and a confidence level of alpha=0.05.

**Results:**

Our results indicated that there were marked regional differences in maternal and child health indicators between these two rural sites, with M'bagne generally performing better across a range of indicators including: immunisation rates, child registration, vitamin A supplementation, deworming, delivery in the presence of a skilled birth attendant, and post-natal care coverage. In Guérou we observed lower rates of fever, diarrhoea, and fast and difficult breathing among children under-five.

**Conclusion:**

Though socio-cultural differences may play a part in explaining some of these observed differences, these alone do not account for the observed differences in maternal and child health indicators. Context-specific activities to overcome barriers to care must be designed to address such rural regional differences if we are to see an improvement across maternal and child health indicators and accelerate progress towards MDGs 4 & 5 in Mauritania.

## Introduction

Despite early indications that Mauritania had made progress in reducing child and infant mortality rates over the last 30 years, projections estimate that improvements have likely stalled since the mid-1990s [1, 2]. Infant mortality rates are estimated to have only marginally decreased from 80 to 75 between 1990 and 2010 [3]. Similarly, under-five (U5) mortality has decreased slightly from 124 to 111 per 1,000 live births in the same period, suggesting that Mauritania is not yet on track towards meeting MDG 4 (reducing child mortality among children under-five by two-thirds) [3, 4]. It remains unclear if this lack of progress is consistent across the nation, or whether it is accounted for by the important discrepancies across rural geographical regions. Moreover, the dearth of reliable health information on the current state of maternal and child health across Mauritania's 13 health departments makes it difficult to identify current gaps and adapt international programmes to meet local needs.

### Child Health

Acute respiratory infections, malaria, and diarrhoeal diseases remain among the leading causes of child morbidity and mortality in Mauritania [5, 6]. At the time of the most recent multi-cluster indicator survey (MCIS3), conducted in 2007, 7% of children under-five reportedly had rapid and difficult breathing two weeks prior to the survey [2]. Likewise, 18% of children under-five were reported to have had a fever at least once in the two weeks preceding the survey; 57% of households owned a mosquito net; and 12% of households were in possession of an insecticide treated net (ITN) [2]. Diarrhoea affected approximately one-fifth (22%) of children under-five, and a more recent study conducted in Nouakchott reported a 52% diarrhoea risk due to poor basic hygiene [7]. Only 35.8% of children aged 12-23 had received all recommended vaccines as part of the Ministry of Health's *Programme Élargi de Vaccination* (Expanded Programme on Immunisation) and one in five children (20%) between the ages of 6-59 months had never received a Vitamin A supplement [2]. In 2007, just over half of children (56%) had had their birth registered [2].

Though the underlying causes of malnutrition are complex, insufficient nutritional food intake and an increased incidence of infection both play an important role [8]. In 2007, 27% of children under-five were considered stunted, one in five children had not received an adequate daily food intake, and 12% were considered underweight [2].

### Delivery and Obstetric Services

The maternal mortality rate for Mauritania has been reported as 690 deaths per 100,000 live births [2], and has been adjusted based on more recent estimates to be 550 per 100,000 [3]. Maternal deaths are most commonly due to direct obstetric causes such as puerperal sepsis, haemorrhage, and hypertensive disease of pregnancy [9, 10]. Mauritania is estimated to have had a 72% prevalence of female genital cutting 2012 [11], which increases the likelihood of delivery complications and neonatal death [12]. Nationally, an average of 48.4% of women gave birth in a health centre within two years of the last MCIS3 in 2007 [2]. Past research has shown large inequalities in access to obstetric care and levels of maternal mortality in urban and rural West Africa [13].

Deliveries attended by skilled birth attendants (SBA) such as nurses, doctors, or midwives, in hygienic conditions, can significantly reduce the risk of complications known to cause death or serious illness in both mothers and their children [14–21]. As a result, this is one of the primary means advocated by the World Health Organisation (WHO) to reduce maternal mortality [22]. In the two years preceding the most recent MCIS3 in 2007, only 61% of live births were attended by a skilled provider [2]. A traditional birth attendant (either trained or untrained) assisted 21% of deliveries, and a worrying 7% of all deliveries reportedly took place without any assistance whatsoever [2]. In the capital Nouakchott, where there is greater access to obstetric care [23], skilled birth attendants reportedly assisted 94% of deliveries [2].

### Antenatal and Postnatal Care

Encouragingly, 81% of women reported accessing antenatal care (ANC) services with a qualified health care provider at least once during their most recent pregnancy [2]. High access to antenatal care has been attributed to the existence of multiple access points, relatively low cost and a more flexible time period for seeking care [24]. In 2007, only 16% of women reported making the recommended 4 antenatal visits and, worryingly, 91% of women in rural areas who delivered at home never received any post-natal care (PNC) following the delivery of their last-born child [2].

### Rationale

Despite the Ministry of Health's *Paquet minimum d'activités* (Minimum Package of Activities) committing to the promotion of health centre and community-based maternal and child health activities across all 13 *wilayat* (regions) [25], maternal and child health coverage still varies greatly across geographic areas. More research is therefore required to draw attention to inequities in service provision, identify current gaps and establish strategies to increase coverage amongst rural populations. The purpose of this paper is therefore to provide updated information on maternal and child health indicators across two rural health departments in Mauritania: Guérou and M'bagne. Guérou is located in the *wilaya* of Assaba in Mauritania's Central Zone, and M'bagne is located approximately 400km south of the capital Nouakchott, on the north banks of the Sénégal River in the *wilaya* of Brakna. Socio-culturally, there are marked differences between both rural populations, with M'bagne being home to a more negro-African influence compared to the more Arab influences in Guérou.

## Methods

The secondary data analysed for this paper were collected as part of a baseline assessment for a maternal and child health programme being implemented in both departments as part of World Vision Ireland's Access to Infant and Maternal Health Programme (AIM-Health).

### Sampling

The baseline exercise employed a cross-sectional household survey and used a two-stage probability sampling method to obtain a sample of the population in each parameter. Village lists were obtained for the rural departments of Guérou and M'bagne, and the probability of a village being selected was set as proportional to the number of households within that village. In Guérou, 48 of the 53 villages were visited and questionnaires were conducted across a sample of 397 households. In M'bagne, a total of 39 out of 46 villages were visited and questionnaires were conducted across a sample of 393 households. Sample size was calculated assuming a confidence level of alpha = 0.05.

In the second stage of sampling, village leaders led field teams to the village centre where a pen was spun to determine the field team's walking direction. A random number generation table was subsequently used to decide which household was to be visited first. Field teams were then instructed to proceed to the next house, until the intended number of household surveys from that village had been met.

### Survey Tool

The survey tool was developed in consultation with local Ministry of Health representatives and with the assistance of maternal and child health experts within the World Vision Partnership. 30 *Agents de santé communautaire* (community health workers, or CHWs) were selected from both Guérou and M'bagne by their communities to participate in the five-day household survey training, hosted by experienced staff from neighbouring health centres. Though the questionnaire was printed in French, training was conducted in a mixture of French, Wolof, and Hassaniya. CHWs were permitted to conduct the interview in whichever language they felt best suited the household. Data collection took place from October-November 2011 and was carried out by World Vision Ireland and World Vision Mauritania.

### Exclusion and Inclusion Criteria

To be considered for analysis the data had to have been collected from an eligible household. The household was defined in terms of any people who were co-resident and shared common cooking arrangements, and were able to recognise one person as the head of household [26]. To be considered eligible, a household had to contain at least one child under the age of 60 months and/or a pregnant woman. Interviews were primarily conducted with the child's primary caregiver, defined as the person who was, “primarily responsible for the health, safety and comfort of that child.”

### Ethical Considerations

Informed written consent was obtained from all participants. If the participant was illiterate, signatures were obtained in the form of a fingerprint using an inkpad. Permission for the Centre for Global Health, Trinity College Dublin to use the de-identified baseline data for secondary analysis was obtained from both World Vision Ireland and World Vision Mauritania, and ethical approval was obtained from the Health Policy and Management/Centre for Global Health Research Ethics committee, Trinity College Dublin.

### Data Analysis

Prior to data analysis, sample characteristics were compared to ensure homogeneity of pregnant women and children under-five in both Guérou and M'bagne. Age and education levels were compared for pregnant women and gender, and ages were compared for children under-five. Data was subjected to descriptive analysis in SPSS Statistics 17 (Release Version 17.0.0), and Pearson's Chi-Squared test, assuming a binomial distribution, was used to test for significant differences. All data was analysed using a confidence level of alpha = 0.05.

For child health indicators, respondents were asked to report whether or not there was an incidence of diarrhoea, cough and rapid and/or difficult breathing, and/or fever for each child under-five in that household over the two weeks preceding the survey. Whether or not the children were reportedly treated at a health centre within 24-hours of the occurrence of this illness was also recorded. Caretakers were also asked whether each child under-five had slept under a mosquito net during the night preceding the survey.

Enumerators verified immunisations for children between the ages of 12-23 months by checking the child health card. Aligned to the guidelines developed by the World Health Organisation (WHO) and adopted by the *Ministère de Santé et des Affaires Sociales* in Mauritania, children were considered fully immunised when they had received the tuberculosis (BCG) vaccine, three doses of both the Pentavalent (Penta) and polio (OPV) vaccines, and a measles vaccination. Health cards for children over the age of 6 months were also verified for the receipt of Vitamin A supplementation in the last 6 months. Health cards were also verified for children over the age of 12 months for the presence of deworming in the last 6 months. Birth registrations were recorded by checking for the presence of a birth certificate. Mid-Upper Arm Circumference (MUAC) measurements were also taken to assess malnutrition among children under-five in both areas. Children who measured as yellow or red were considered to be malnourished.

For each child under-five, the following information was collected: child delivered in a health centre or not; birth attended by SBA or not; birth attended by traditional birth attendant (TBA) or not. At the time of data collection, ANC visits were recorded for pregnant women by checking the maternal health card and recording the number of completed visits out of the minimum recommended 4 clinics. As part of ANC, pregnant women were asked to correctly identify where they could access prevention of parent to child transmission of HIV (PPTCT) services, whether or not they had slept under a mosquito net during the night preceding the survey, and whether they were currently taking iron and folic acid supplements. For each child under-five, whether or not their mother had received at least one of the recommended two PNC visits within 24 hours of their birth was also recorded.

## Results

### Characteristics of the Sample

A total of 1505 children under the age of 5 and 274 pregnant women across 790 households in Guérou and M'bagne were included in the sample for analysis. Pearson's Chi-Squared test of significance revealed no significant differences in gender (X2(1) = 0.074, p > 0.05) or age category (X2(4) = 0.957, p > 0.05) for children under-five across both locations. Similarly, no significant differences were found for age category (X2(2) = 3.960, p > 0.05) or education level (X2(2) = 3.695, p > 0.05) for pregnant women across the two areas. The results of the comparison of sample characteristics are depicted in Table 1.

**Table 1:** Sample characteristics

	Guérou	M'bagne	Chi-Squared (degrees of freedom)	p-value
**Children under-five**				
**Gender**	n= 631	n = 874		
Male	51.3%	52.1%	0.074 (1)	0.785
Female	48.7%	47.9%		
**Age**	n= 627	n= 873		
0 to 11 months	16.9%	17.2%	0.957 (4)	0.916
12 to 23 months	17.4%	17.0%		
24 to 35 months	20.4%	22.2%		
36 to 47 months	22.3%	22.1%		
48 to 59 months	23.0%	21.5%		
**Pregnant Women**				
Age	n= 96	n= 178		
14 to 18 years	18.7%	10.7%	3.960 (2)	0.138
19 to 49 years	81.3%	88.8%		
50 years or older	0.0%	0.6%		
Education Level	n= 95	n= 175		
None	52.6%	64.6%	3.695 (2)	0.158
Some Primary	36.8%	28.0%		
Some Secondary	10.5%	7.4%		

### Child Health

Results indicate that there were marked differences in the prevalence of symptoms for common childhood illnesses across the two locations and that M'bagne had a higher prevalence of illness than Guérou. Significant differences between the two sites were found in the prevalence of symptoms for the following common childhood illness indicators: diarrhoea (X2(1) = 9.441, p < 0.05), fever (X2(1) = 6.534, p < 0.05), and cough and/or fast and difficult breathing (X2(1) = 5.968, p < 0.05) (Table 2). Less than half (45.9%, (38.8, 53.1)) of all ill children in M'bagne (n = 185) had been brought to a health centre for treatment within 24-hours, dropping to just over a quarter (27.8%, (18.9,36.8)) in Guérou (n = 97), with a statistically significant difference (X2(1) = 8.718, p < 0.05) between the two areas.

**Table 2:** Common childhood illness prevalence in children under-five

Illness	Guérou	M'bagne	Chi-Squared (degrees of freedom)	p-value
Diarrhoea	5.4%	9.7%	9441 (1)	0.002
Fever	11.4%	16.0%	6.534 (1)	0.011
Acute Respiratory Infection*	5.2%	8.5%	5.968 (1)	0.015

* ARI is defined as having fast and difficult breathing with or without the presence of a cough

Rates of immunisation coverage were significantly higher in M'bagne (n = 147), with 95.9% (2.7 - 99.1) of children aged 12-23 months having completed the full immunisation schedule, compared to only three quarters (76.4%, 68.3 - 84.5) of children in Guérou (n = 106). Analysis yielded a significant difference between the two areas (X2(1) = 21.789, p < 0.05). This pattern was consistent throughout the individual vaccines of the full immunisation schedule: BCG (X2(1) = 15.751, p < 0.05), measles vaccine (X2(1) = 23.865, p < 0.05), a minimum of three OPV (X2(1) = 17.579, p < 0.05), and all three doses of Penta vaccines (X2(1) = 13.134, p < 0.05) (Table 3). Differences were also found for the prevalence of children between the ages of 6-60 months having received at least one vitamin A supplement and children between the ages of 12-60 having received at least one deworming tablet in the last six months, respectively, with significantly greater coverage for both reported indicators in M'bagne (Table 4).

**Table 3:** Immunisation coverage in children aged 12-23 months

Immunisation (all)	Guérou	M'bagne	Chi-Squared (degrees of freedom)	p-value
BCG	84.3%	100.0%	15.751 (1)	<0.001
OPV 0	88.8%	98.6%	11.644 (1)	0.001
OPV 1	87.7%	100.0%	19.005 (1)	<0.001
OPV 2	85.7%	98.0%	13.846 (1)	<0.001
OPV 3	84.6%	98.0%	15.500 (1)	<0.001
At least 3 Oral Polio Vaccines	84.9%	98.6%	17.579 (1)	<0.001
Penta 1	88.6%	98.6%	11.833 (1)	0.001
Penta 2	85.7%	98.6%	16.265 (1)	<0.001
Penta 3	84.8%	98.0%	15.303 (1)	<0.001
All 3 Penta	84.8%	97.3%	13.134 (1)	<0.001
Measles	77.9%	97.3%	23.865 (1)	<0.001
Fully immunised*	76.4%	95.9%	21.789 (1)	<0.001

**Table 4:** Other health factors for children under-five

*Other Health Factors*	Guérou	M'bagne	Chi-Squared (degrees of freedom)	p-value
Vitamin A*	91.6%	98.2%	31.862 (1)	<0.001
Dewormed^	85.0%	96.9%	59.752 (1)	<0.001
Slept under malarial net	42.6%	88.9%	362.530 (1)	<0.001
Registered birth˜	46.6%	77.1%	147.882 (1)	<0.001

* Children over 6 months who had received any vitamin A supplement

^ Children over 12 months who had been dewormed

˜ Children under 5 with a birth certificate

Less than half (46.6%) of children under-five in Guérou (n = 625, 42.6 - 50.5) had their birth registered compared to 77.1% in M'bagne (n = 873, 74.3 - 79.9), with significant differences found between the two areas (X2(1) = 147.882, p < 0.05). A reported 42.6% of children under-five in Guérou (n = 615, 38.7 - 46.5) had slept under a mosquito net throughout the night preceding the survey. This was found to be significantly less than the reported 88.9% of children under-five who slept under a mosquito net in M'bagne (X2(1) = 362.530, p < 0.05). MUAC measurements found 6.9% (n = 793, 5.2 - 8.7) of children in M'bagne and 7.1% (n = 622, 5.1 - 9.1) of children in Guérou as either yellow or red. A reading of yellow was significantly more common in M'bagne (X2(2) = 16.755, p < 0.05), however there were no significant differences in overall malnutrition rates across the two regions.

### Delivery and Obstetrics Services

In Guérou, 60.0% (n = 630, 56.2 - 63.8) of children under-five at the time of the survey had reportedly been delivered in a health centre compared to 67.5% of children under-five (n = 871, 64.4 - 70.6) reportedly delivered in a health centre in M'bagne, with a statistically significant difference (X2(1) = 8.985, p < 0.05) identified between the two areas. Similarly, significantly fewer children under-five (63.7%) were delivered by a skilled birth attendant in Guérou (n = 630, 59.9 - 67.4) than were delivered (70.4%) by a SBA in M'bagne (n = 871, 67.3 - 73.4) (X2(1) = 7.550, p < 0.05). In Guérou, 38.6% (n = 628, 34.9 - 42.5) of children under-five had their birth assisted by a traditional birth attendant (TBA). Comparing this to the 21.1% of children under-five (n = 873, 18.4 - 23.8) delivered by a TBA in M'bagne, we found a statistically significant difference (X2(1) = 55.694, p < 0.05) between the two areas. The rates of children under-five who were delivered without either a traditional or skilled birth attendant was minimal in Guérou (0.8%, n = 630, 0.1 - 1.5), but represented over 1 in 10 children in M'bagne (11.6%, n = 873, 9.4, 13.7), with a statistically significant difference (X2(1) = 64.818, p < 0.05) between the two areas.

### Antenatal and postnatal care

The majority of pregnant women interviewed had attended at least one ANC visit throughout their pregnancy. Significant differences were found in the numbers of pregnant women attending at least one ANC in Guérou (n = 89, 61.3 - 80.2) when compared to M'bagne (n = 162, 83.3 - 93.2). When asked where one might go to access PPTCT, more respondents answered correctly in Guérou compared to M'bagne. Fewer pregnant women in Guérou (n = 95, 20.3 - 38.6) had slept under a mosquito net the night preceding the survey compared to M'bagne (n = 179, 83.6 - 93.0). At the time of the survey, 68.2% (n = 88, 58.5 - 77.9) of pregnant women were taking folic acid and 71.3% (n = 87, 61.8 - 80.8) were taking iron sulphate supplements in Guérou, with 87.6% (n = 169, 79.2 - 90.1) taking folic acid and 87.4% (n = 175, 79.2 - 89.9) taking iron sulphate supplements in M'bagne (Table 5).

**Table 5:** Health factors in pregnant women

	Guérou	M'bagne	Chi-Squared (degrees of freedom)	p-value
**ANC Visits**				
At least one ANC visit	70.8%	88.3%	11.935 (1)	0.001
No ANC visits	28.2%	11.7%		
**Knowledge**				
Where to access PPTCT*	68.6%	25.5%	6.177 (1)	0.013
**Behaviour**				
Slept under malarial net	29.5%	88.3%	98.402 (1)	<0.001
Folic Acid supplement	68.2%	87.6%	13.756 (1)	<0.001
Iron Sulfate supplement	71.3%	87.4%	10.073 (1)	0.002

* Correctly identified the health centre as a place to access prevention of parent to child transmission

Only a fifth (21.0%) of children under-five in Guérou (n = 625, 17.8 - 24.2) were recorded as having had any PNC visit compared to over half (56.3%) in M'bagne (n = 872, 53.0 - 59.6), with a statistically significant difference between the two areas (X2(1) = 187.298, p < 0.05).

It follows that women who deliver in a health centre would automatically receive their first PNC visit before being discharged. In Guérou, 31.6% of children born in a health centre received at least one PNC visit. In comparison, 5.2% of children who were not born in a health centre received at least one PNC. In M'bagne, 65.9% of children born in a health centre received at least one PNC visit, compared to 37.1% of children who were not born in a health centre.

## Discussion

Our results show that there are marked regional differences in maternal and child health indicators between the two rural sites, with M'bagne generally performing better across most of the indicators examined. A summary of these findings is depicted in Table 6.

**Table 6:** Summary of child and maternal health factors

	Guérou	M'bagne	Chi-Squared (df)	p-value
**Child Health**				
Illness Prevalence				
Diarrhoea	5.4%	9.7%	9441 (1)	0.002
Fever	11.4%	16.0%	6.534 (1)	0.011
ARI	5.2%	8.5%	5.968 (1)	0.015
Treatment of Illness				
HC for illness	27.8%	45.9%	8.718 (1)	0.003
HC for diarrhoea	21.2%	40.0%	3.701 (1)	0.054
HC for fever	31.4%	50.0%	6.509 (1)	0.011
HC for ARI	39.4%	46.4%	0.442 (1)	0.506
Immunisations				
Fully immunised	76.4%	95.9%	21.789 (1)	<0.001
BCG	84.3%	100.0%	15.751 (1)	<0.001
3 OPV	84.9%	98.6%	17.579 (1)	<0.001
3 Penta	84.8%	97.3%	13.134 (1)	<0.001
Measles	77.9%	97.3%	23.865 (1)	<0.001
Other Health factors				
Vitamin A	91.6%	98.2%	31.862 (1)	<0.001
Dewormed	85.0%	96.9%	59.752 (1)	<0.001
Birth certificate	42.6%	88.9%	362.530 (1)	<0.001
Slept under a net	46.6%	77.1%	147.882 (1)	<0.001
MUAC level				
Red or Yellow	7.1%	6.9%	0.010 (1)	0.919
Green	92.9%	93.1%		
**Delivery and Obstetrics**				
Delivery Location				
Delivered in HC	60.0%	67.5%	8.985 (1)	0.003
Delivery Circumstances				
Delivered by SBA	63.7%	70.4%	7.550 (1)	0.006
Delivered by TBA	38.6%	21.1%	55.694 (1)	<0.001
Delivered without SBA or TBA	0.8%	11.6%	64.818 (1)	<0.001
**ANC and PNC**				
ANC Visits				
At least one ANC	70.8%	88.3%	11.935 (1)	0.001
No ANC	28.2%	11.7%		
Knowledge				
Where to access PPTCT	68.6%	25.5%	6.177 (1)	0.013
Behaviour				
Slept under net	29.5%	88.3%	98.402 (1)	<0.001
Iron Sulphate	68.2%	87.6%	13.756 (1)	<0.001
Folic Acid	71.3%	87.4%	10.073 (1)	0.002
PNC Visits				
At least one PNC	21.0%	56.3%	187.298 (1)	<0.001
No PNC	79.0%	43.7%		
PNC and HC delivery				
HC & PNC	18.9%	44.4%	107.827 (1)	<0.001
HC & no PNC	41.0%	23.0%		
Not HC & PNC	2.1%	12.1%	78.748 (1)	<0.001
Not HC & no PNC	38.1%	20.5%		

MUAC: Mid Upper Arm Circumference; HC: Health Center; SBA: Skill birth attendant; TBA: traditional birth attendant; ANC: Ante natal care; PNC: Prenatal consultation

### Characteristics of the Sample

Both maternal age and education have been identified as important factors associated with better maternal and child health outcomes [27–32]. The failure to find statistically significant differences between both the age and education levels of pregnant women in each group suggests that the two samples of pregnant women were comparable for the purpose of further analysis and that variations in education or age levels were not responsible for observed differences between the two samples. Similarly, variations in age and gender for children under-five were not responsible for observed differences across the two samples of children.

### Child Health

The prevalence of fever reported in M'bagne (16%) is consistent with what was published in the MCIS3 for the *wilaya* of Brakna (15.2%) [2]. Our results indicate that there is a significant difference between the two regions, with Guérou demonstrating a lower prevalence of fever in children under-five. The rates of suspected ARIs in Guérou (5.2%) and M'bagne (8.5%) are consistent with the national prevalence reported in the MCIS3 (6.5%). Comparing the two departments, our results found greater reported prevalence of suspected ARIs in M'bagne. The rate of caretakers accessing health centres within 24 hours in response to an identified danger sign (45.9%) is consistent with the prevalence of children being brought to the health centre within 24-hours for suspected pneumonia nationally as reported by the MCIS3 (44.5%). Furthermore, our results show that the prevalence of caretakers taking a child to health centre within 24-hours of identifying a potential illness was significantly greater in M'bagne (45.9%) compared to Guérou (27.8%). One potential explanation for these results may be the failure of caretakers to recognise these specific symptoms in children. Educating households about how to prevent and identify childhood illness danger signs and increasing access to primary caregivers in health centres are both important in order to reduce delays in children being treated for common childhood illnesses.

The implementation of successful national immunization programmes, such as Mauritania's *Programme Élargi de Vaccination*, may have in part contributed to the important progress of vaccination coverage in Mauritania. Significant differences in completed vaccination schedules for children between the ages of 12-23 months indicate greater vaccination coverage in M'bagne. Similarly, our results indicate significantly greater prevalence of children aged 6-59 months having received Vitamin A in M'bagne in the 6 months preceding the survey. Better immunisation rates and greater prevalence of Vitamin A supplements are also consistent with the greater observed prevalence of children aged 12-59 months having received a deworming tablet in M'bagne. Differences between the two sites suggest that there is a need to focus greater resources and increase access to supplementation coverage and vaccination programmes in Guérou. We found no significant differences in rates of malnutrition between Guérou and M'bagne.

### Delivery and Obstetric Services

Our results indicate that the prevalence of children born in a health centre and in the presence of a skilled birth attendant was significantly higher in M'bagne. The greater prevalence of children under-five born in a health centre in M'bagne is consistent with a higher prevalence of children under-five in M'bagne born in the presence of a SBA and with a lower number of children born in the presence of a TBA. The prevalence of children born in health centres and in the presence of an SBA was higher than those reported for both Assaba and Brakna during the most recent MCIS3. Originally only available in Nouakchott, early evaluations of the *Forfait Obstétrical* (Obstetric Risk Insurance) programme reported an increase in the rates of hospital and post-natal care for pregnant women living in the capital [23]. One hopes the success of this programme will continue as it starts to extend the insurance option to more rural areas across Mauritania. Further inquiry is required to better understand the higher rates of children born in the absence of either a TBA or a relative in M'bagne.

### Antenatal and Postnatal Care

Participants in M'bagne reported better use of folic acid and iron sulfate supplements in pregnant women compared to Guérou. Important information related to iron and folic acid supplements, malaria prevention, HIV and the possible transmission to a child should be reiterated during routine ANC visits by health centre workers. Though the prevalence rate of HIV among pregnant women in Mauritania remains relatively low (between 0.1 and 1.48), few women interviewed from M'bagne correctly identified the health centre as a place to access prevention of parent to child transmission of HIV [33]. More sensitization is necessary to inform women and key household health decision makers regarding where pregnant women can access PPTCT services.

The prevalence of women having visited at least one ANC is consistent with past research demonstrating higher health facility attendance for antenatal services compared to labour and delivery services [34]. The challenge therefore remains to ensure that women are accessing the appropriate information during ANC visits, that they are attending them at appropriate times, and that they complete all four of the minimum recommended visits prior to their estimated delivery date.

Whereas M'bagne seems to have shown progress in the prevalence of pregnant women sleeping under mosquito nets (88.3%), the low prevalence in Guérou (29.5%) remains consistent with the most recent national average of 3.2% [1]. Despite the survey having taken place towards the end of the rainy season in Nouakchott, the reasons why mosquito nets were not in use by pregnant women in Guérou are unclear [6]. Further investigation is necessary to establish whether this is due to insufficient resources and/or whether behaviour change is necessary to increase the number of pregnant women sleeping under mosquito nets.

The greatest gap in the continuum of care occurs during the first week after childbirth, when the risk of both maternal and newborn deaths is most likely [35]. It is therefore important that households in both regions have access to PNC. This is especially true in Guérou where our results show a worryingly low uptake of PNC services after delivery.

## Conclusion

Though socio-cultural differences exist between Guérou and M'bagne, these alone do not account for the observed regional differences in maternal and child health indicators. Other factors such as geographical inaccessibility, limitations in human resources for health, religious ideologies, and the presence and activities of existing NGOs in the area must also be considered. Insufficient progress in improvements in maternal and child health suggests that there is an important need for the continued support of effective actions and for implementing new programmes aimed at improving child health in rural geographic areas in Mauritania. Context-specific activities to overcome barriers to care must be designed to address such rural regional differences if we are to see an improvement across maternal and child health indicators and accelerate progress towards MDGs 4 & 5 in Mauritania.
